# Migrating to Long-Read Sequencing for Clinical Routine *BCR-ABL1* TKI Resistance Mutation Screening

**DOI:** 10.1177/11769351221110872

**Published:** 2022-07-15

**Authors:** Wesley Schaal, Adam Ameur, Ulla Olsson-Strömberg, Monica Hermanson, Lucia Cavelier, Ola Spjuth

**Affiliations:** 1 Department of Pharmaceutical Biosciences, Uppsala University, Uppsala, Sweden; 2 Pincer Bio AB, Uppsala, Sweden; 3 Department of Immunology, Genetics and Pathology, Uppsala University, Uppsala, Sweden; 4 Department of Medical Sciences, Uppsala University Hospital, Uppsala, Sweden

**Keywords:** Long-read sequencing, SMRT sequencing, drug resistance, chronic myeloid leukemia, BCR-ABL1, CML, mutation screening

## Abstract

**Objective:**

The aim of this project was to implement long-read sequencing for BCR-ABL1 TKI resistance mutation screening in a clinical setting for patients undergoing treatment for chronic myeloid leukemia.

**Materials and Methods:**

Processes were established for registering and transferring samples from the clinic to an academic sequencing facility for long-read sequencing. An automated analysis pipeline for detecting mutations was established, and an information system was implemented comprising features for data management, analysis and visualization. Clinical validation was performed by identifying BCR-ABL1 TKI resistance mutations by Sanger and long-read sequencing in parallel. The developed software is available as open source via GitHub at https://github.com/pharmbio/clamp

**Results:**

The information system enabled traceable transfer of samples from the clinic to the sequencing facility, robust and automated analysis of the long-read sequence data, and communication of results from sequence analysis in a reporting format that could be easily interpreted and acted upon by clinical experts. In a validation study, all 17 resistance mutations found by Sanger sequencing were also detected by long-read sequencing. An additional 16 mutations were found only by long-read sequencing, all of them with frequencies below the limit of detection for Sanger sequencing. The clonal distributions of co-existing mutations were automatically resolved through the long-read data analysis. After the implementation and validation, the clinical laboratory switched their routine protocol from using Sanger to long-read sequencing for this application.

**Conclusions:**

Long-read sequencing delivers results with higher sensitivity compared to Sanger sequencing and enables earlier detection of emerging TKI resistance mutations. The developed processes, analysis workflow, and software components lower barriers for adoption and could be extended to other applications.

## Introduction

Next-generation sequencing (NGS) technologies are a dramatic improvement over traditional Sanger sequencing in terms of throughput, cost, and accuracy.^1^ The parallel nature of NGS can transform large projects into routine activities.^2^ With all its advantages, NGS can have difficulties with highly-repetitive or other problematic regions. It can be difficult to stitch together a consensus sequence from very similar short read fragments.^3^ Long-read single-molecule sequencing (LR-SMS) ameliorates this problem by producing fragments long enough to unambiguously read through difficult regions.^4,5^ Because of these advantages, LR-SMS is increasingly being used for human medical research, for example to identify previously uncharacterized structural variation, determine the clonal distribution of mutations, and to sequence through repetitive regions.^6^ However, there is today a lack of established workflows and analysis tools which focus on LR-SMS for routine clinical diagnostics.

Chronic Myeloid Leukemia (CML) is a well-studied disease. Efficient drug treatments using tyrosine kinase inhibitors (TKIs) are available, but some patients develop drug resistance. This resistance can in many cases be explained by point mutations in the *BCR-ABL1* fusion gene, which is the intended target for the TKI’s. BCR-ABL1 TKI resistance is also relevant for the related disease Acute Lymphoblastic Leukemia (ALL).^7^ The multi-resistant substitution T315I is of particular importance for clinical investigations, but other nucleotide substitutions in BCR-ABL1 can also confer resistance. In other clinical cases, the reason for resistance is still unknown.^8^

As part of the treatment process, Sanger sequencing has traditionally been used to identify T315I and other mutations. This has worked reasonably well but since the sensitivity to detect low-frequency mutations using Sanger sequencing is 15% to 20%,^9^ this is limiting the detection of emerging drug-resistant mutations.^2^ Short read NGS makes it possible to detect mutations at lower frequency, but does not generally provide information about clonal composition of co-occurring mutations since the sequence information is broken into small pieces. LR-SMS resolves all of these issues and when obtaining high-accuracy reads, which are currently enabled by PacBio circular consensus sequencing,^10^ false positive mutations due to sequencing errors can essentially be removed.^11^

Our previous work demonstrated that LR-SMS can be applied to studying BCR-ABL1 mutations in CML patients.^12^ Moreover, a dilution series was performed demonstrating that low frequency mutations (<1%) are detectable.^12^ As a relatively new technology, there are fewer bioinformatics packages available for LR-SMS analysis and no turn-key solutions. This presents difficulties for clinical organizations or any organization with established routines where this expertise is not available. To facilitate translation of LR-SMS into clinical applications, there is a need for processes as well as informatics and decision support systems to assist with organizing and automating the analysis work needed for LR-SMS.

Here we illustrate how these challenges have been overcome at Uppsala University Hospital for an application in BCR-ABL1 TKI resistance mutation screening. We present how this transition was made via parallel studies using Sanger and PacBio single molecule real-time (SMRT) sequencing with summary statistics from 39 patients. We also resolved a number of logistical issues such as long turnaround for the analyses, inefficient communication through email and written reports with little tractability to samples from the same or other patients. To this end, a web-based system to organize and automate data management, analysis, and visualization was developed.

## Methods

### Patient samples

Data were collected from 39 samples analyzed through Uppsala University Hospital. The patients were being treated for Chronic Myeloid Leukemia (CML, 36 samples) or Acute Lymphocytic Leukemia (ALL, 3 samples) and suspected to be resistant to the therapy. Treatments, responses, and disease status varied.

### Ethics statement

This study was performed in accordance with the Declaration of Helsinki. The Ethical Committee at Uppsala University, Dnr 2014/233, approved this study. Written informed consent was obtained from the patients.

### RNA extraction and cDNA synthesis

RNA was extracted from peripheral blood or bone marrow samples using a TRIzol^®^ (Invitrogen), standard protocol and quantified by NanoDrop™ 2000/2000c Spectrophotometers (Thermo Scientific, MA, USA). cDNA was synthesized using the SMARTer™ PCR cDNA synthesis kit (ClonTech, CA, USA), using 1000 ng total RNA.

### Library preparation and PacBio sequencing

Long range PCR amplification of the BCR-ABL1 transcript was performed using the Clontech Advantage PCR kit as previously described.^12^ The cDNA amplicons underwent end-repair and adaptor ligation to generate SMRTbell™ libraries for PacBio sequencing. SMRTbell™ libraries were quantified using the Qubit assay and library size was confirmed using the Agilent DNA 12000 Kit. Each SMRTbell™ amplicon library was loaded on to 1 SMRT cell and sequenced on the PacBio RS II instrument using C4 chemistry and a 120-minute movie time. Circular consensus sequence (CCS) reads were generated for each sample. The CCS reads in FASTQ format were used as input for the automated analysis pipeline.

### LR-SMS analysis workflow

**Figure 1.**
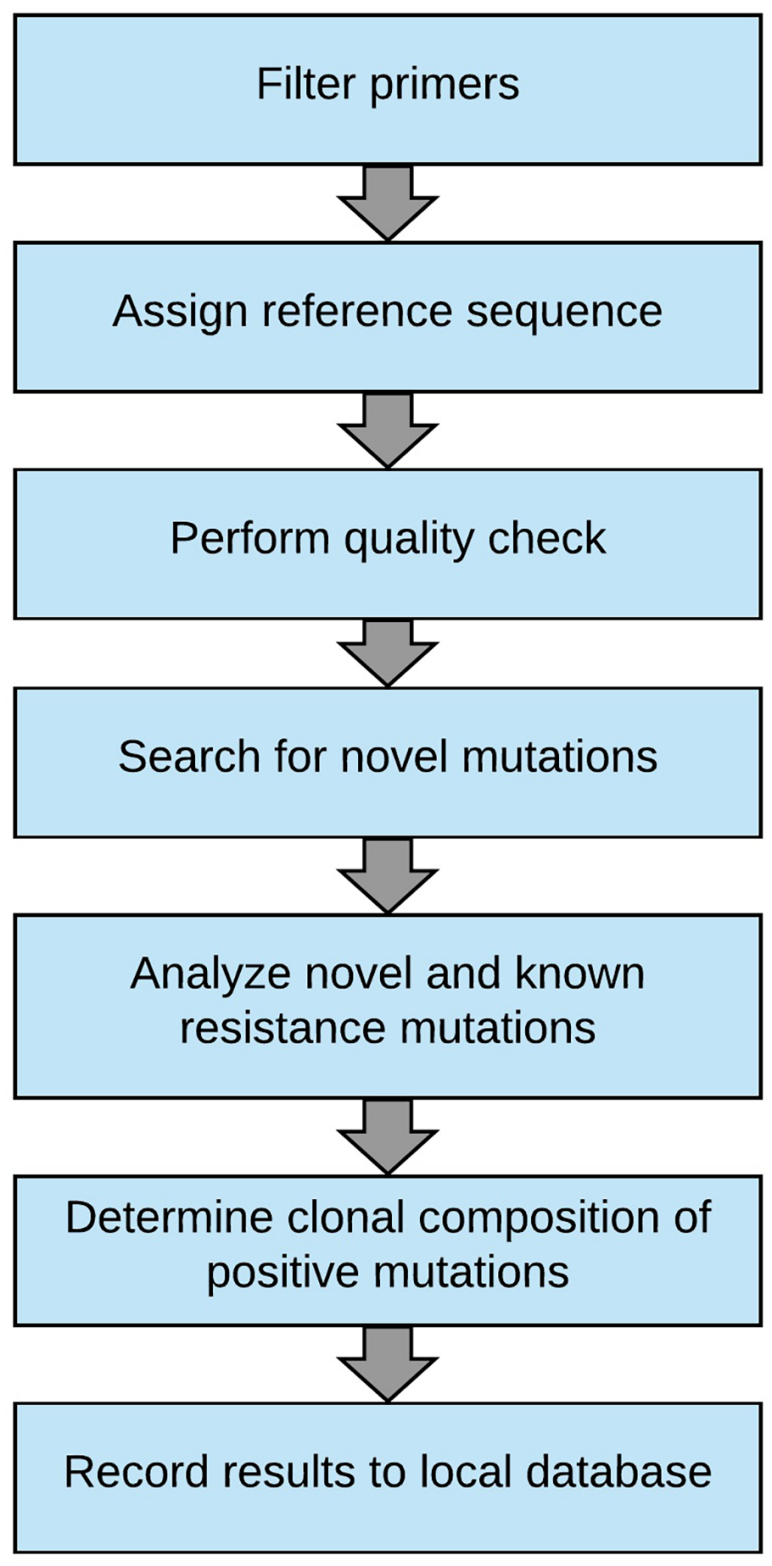
Bioinformatics workflow. The flow chart shows the analysis steps carried out by the automated analysis software. A FASTQ-formatted file is used as input to the analysis pipeline. The individual analysis steps are described in more detail in the Methods section.

An analysis workflow was developed to process PacBio sequencing data according to the general procedure described below and summarized in Figure 1. FASTQ files are automatically processed through a collection of custom scripts written in R and Perl. The analysis workflow is available as Open Source via GitHub (https://github.com/pharmbio/clamp). The analysis can be divided into 7 main steps, described below:

(1) Filter primers. In the first step, all CCS reads are filtered so that only reads having the expected primer sequences both at the 5′ and 3′ ends are kept. In this way, only reads corresponding to the BCR-ABL1 fusion gene are used for further analysis.
(2) Assign reference sequence. Next, the filtered reads are used to determine the main isoform of BCR-ABL1 present in the sample. The reason for this analysis is that there can be some small variation in exon usage between different samples. Once the dominant isoform has been determined, this isoform is assigned as a reference sequence for the subsequent steps.
(3) Quality check. A quality control is performed to make sure the entire sequence is covered by at least 100 reads, to ensure that no mutation sites are missed. If the quality check fails, then the analysis is aborted.
(4) Search for novel mutations. After having passed the quality control, the sample is screened for previously unreported (de novo) mutations. The reads are compared to the reference sequence and all sites with potential nucleotide substitutions are reported.
(5) Analyze novel and known resistance mutations. Next, all previously known and potentially novel TKI resistance mutations in BCR-ABL1 are analyzed and their variant allele frequencies (VAF) determined. The results are compiled into a table.
(6) Determine clonal composition of positive mutations. In cases where least 2 positive mutations are found, an additional analysis is performed to determine the clonal distribution of all mutations in the sample.
(7) Record results in local database. After processing, the raw data files, quality control data, and mutation results are stored in a custom developed information system.

### Information system for long-read sequencing

**Figure 2.**
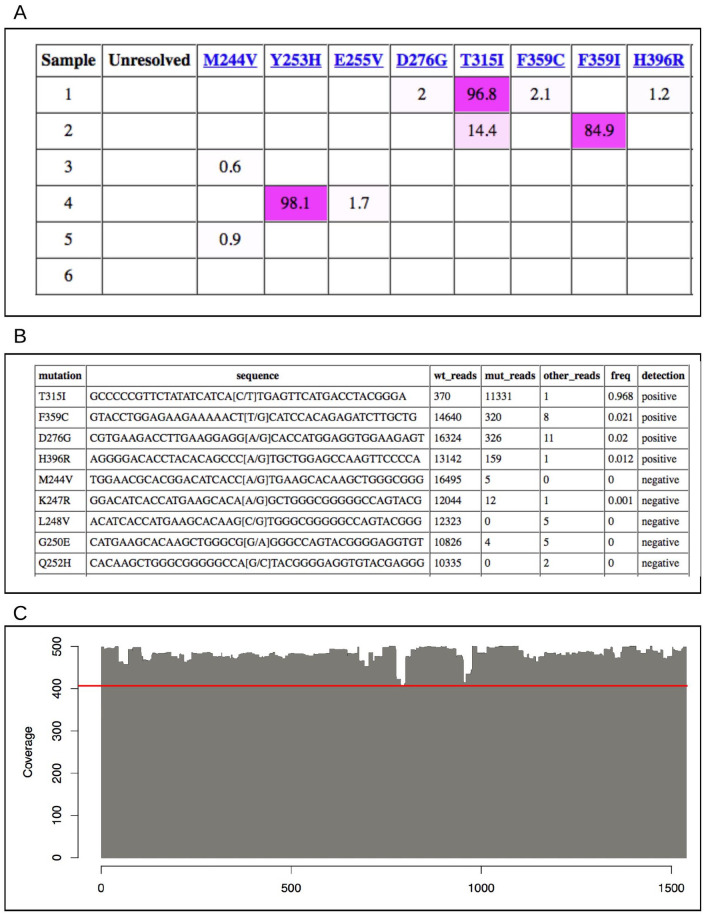
Examples of visualizations from the LR-SMS results interface (excluding some site-specific details). (A) Subset of results from the LR-SMS reporting system. The coloring highlights mutation percentage. Only the mutations found in the current search results are listed. (B) A portion of the mutation report for a sample. Four positive mutations were found in this patient sample (T315I, F359C, D276G, and H396R) while all other mutations were negative. (C) Coverage of reads at each position of the BCR-ABL1 reference sequence of 1 sample. Since each position is covered by at least 400 reads (see red line), we are confident that mutations in all parts of the fusion gene can be efficiently analyzed.

**Figure 3.**
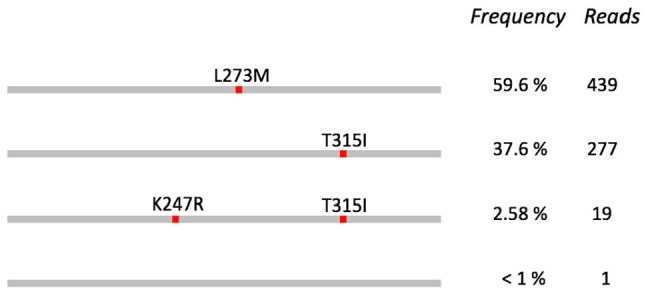
Clonal distribution of Sample 15. Long-read sequencing shows which mutations occur together rather than in separate populations. In this sample, the K257R mutation occurs only with T315I, never in isolation.

An information system was developed to sustain registration of samples of the clinical side, automated analysis of the produced sequencing data, storage of results in a database, and communication of results to clinicians. The database was implemented on top of SQLite and the interfaces consisting of custom Perl scripts are served over a private intranet with Apache HTTPD. Searches can be made through various fields and a summary of the matches is returned (Figure 2A). Details for single samples include a detailed report of mutations (Figure 2B), quality control (Figure 2C), and clonal distribution the co-existing mutations (Figure 3). The highlights of this simple system are the ease of sample report retrieval and the ability to make basic comparisons. The source code for the information system is available as Open Source via GitHub (https://github.com/pharmbio/clamp).

## Results and Discussion

### Implementation in clinic

At the Clinical Genetics Division at Uppsala University Hospital (UUH), TKI resistance mutations have traditionally been detected using Sanger sequencing of the BCR-ABL1 fusion gene. We set out to link UUH to the sequencing facility National Genomics Infrastructure (NGI) at Science for Life Laboratory (SciLifeLab) in Sweden.

**Figure 4.**
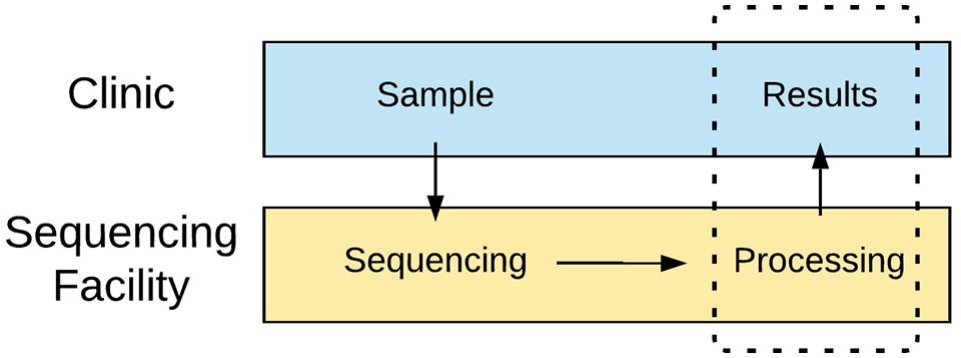
Overview of the implemented workflow from samples to interpretable information. Clinicians send samples to the university for long-read sequencing. After sequencing, technicians upload data to the analysis software for automated processing. Processed data are stored so clinicians can retrieve results through a searchable web form. The area within the dotted line is served by the support system created for this project.

We established a process according to the following: (i) Clinicians register the sample and perform RNA extraction and cDNA synthesis; (ii) Samples are transferred between the hospital and the NGI sequencing facility in Uppsala; (iii) Long-read single molecule sequencing is carried out at the facility; (iv) An automated analysis pipeline is executed; (v) Results are stored in a shared database; (vi) Clinicians are informed of new results and can view and download actionable reports. The process is illustrated in Figure 4.

In a previous dilution series experiment,^12^ we were able to identify BCR-ABL1 TKI resistance mutations with variant allele frequencies (VAFs) as low as 0.5%, which is far below the detection limit for routine Sanger analysis.^9^ Since early detection of emerging mutations is important to guide treatment and to avoid resistance development, this highlights a major advantage of LR-SMS.^13^ Based on our previous work, the cutoff for a positive mutation was set to 0.5%. However, only mutations above 1% were considered relevant by the clinicians to reduce the risk of false positives.

The LR-SMS analysis workflow is simple and automated. Technicians need only to run a single script and upload the resulting files to produce quality control reports and mutation results. Result retrieval by clinicians was similarly simplified over the former practice of exchanging static documents. Feedback from clinicians helped design a data analysis system that they can and want to use. These small changes in procedure helped ease the transition away from Sanger to PacBio SMRT sequencing.

### Validation study

LR-SMS was run in parallel with routine Sanger sequencing for a period of 25 months. During this period, 39 samples from patients not responding to TKI treatment were collected to determine the presence of BCR-ABL1 resistance mutations (Table 1). A total of 17 mutations were found by Sanger in the 39 samples and all of these were detected also by LR-SMS. LR-SMS identified an additional 16 mutations that were not found with Sanger sequencing. Eight of these mutations were detected above the 1% limit. In many of the samples where LR-SMS identified more mutations, those mutations were reported at levels that would be difficult for Sanger to detect.^9^

**Table 1.** Samples studied with mutations detected by Sanger and LR-SMS sequencing. Percentage of mutation occurrence for LR-SMS is shown in parentheses. Occurrences below 1% were not considered for clinical use.

Sample	Sanger	LR-SMS
1	T315I	T315I (96.8), F359C (2.1), H396R (1.2)
2	F359I, T315I	F359I (84.9), T315I (14.4)
3	—	M244V (0.6)
4	Y253H	Y253H (98.1), E255 (1.7)
5	—	M244V (0.9)
6	—	—
7	Y253H	Y253H (99.8)
8	—	—
9	—	—
10	T315I	T315I (99.9)
11	—	—
12	T315I	T315I (50.0), E255V (1.9)
13	—	—
14	T315I, E255K, F359V	T315I (45.7), F359I (23.9), E255K (12.5), H396R (12.4), F359V (4.8)
15	L273M, T315I	L273M (58.1), T315I (41.7), K247R (2.8)
16	T315I	T315I (99.8)
17	—	—
18	—	E450G (0.5)
19	—	—
20	—	—
21	—	—
22	—	—
23	—	—
24	F359I	F359I (91.3), T315I (8.3)
25	—	—
26	—	T315I (0.6), E450G (0.5)
27	—	—
28	—	M472I (0.8)
29	F359V	F359V (99.9), D276G (0.7)
30	—	—
31	—	—
32	—	—
33	L298V, E255K	L298V (33.6), E255K (29.6)
34	—	—
35	—	E450G (0.8)
36	—	—
37	—	—
38	—	—
39	—	—

In 21 samples, no mutations were reported by either Sanger or LR-SMS. Of the 18 samples which did show mutations, 5 had complete agreement between the methods. Of the remaining 13 samples, LR-SMS found more mutations than Sanger. No mutations identified by Sanger were missed by LR-SMS.

Samples 4 and 7 were found by both methods to include mutation Y253H, which is associated with imatinib resistance.^8^ LR-SMS reported this mutation as strongly dominating (99.8% and 98.1%, respectively). In addition, LR-SMS detected a modest amount (1.7%) of mutation E255V in sample 4. This mutation, also associated with imatinib resistance, was not identified by Sanger, probably due to its low prevalence.

In Sample 15, both methods identified mutations L273M and T315I but LR-SMS also reported the rare mutation K247R. T315I is the well-known “gatekeeper” mutation associated with imatinib and secondary resistance.^14^

One of the benefits of long-read sequencing is that phasing of mutations separated by several kilo bases becomes a trivial task, whereas this information is more difficult to obtain from other sequencing technologies. This is exemplified in Figure 4 for Sample 15. The mutations L273M and T315I occur in distinct molecules, but there is also a subset of molecules that contain both T315I and K247R. This type of information can help us understand the clonal evolution of cancer cells in a patient during course of therapy. The clinical significance of this information may not yet be known, but the ability to routinely analyze the clonal distribution of BCR-ABL1 mutations could potentially lead to improved therapeutic decisions.^15^

**Figure 5.**
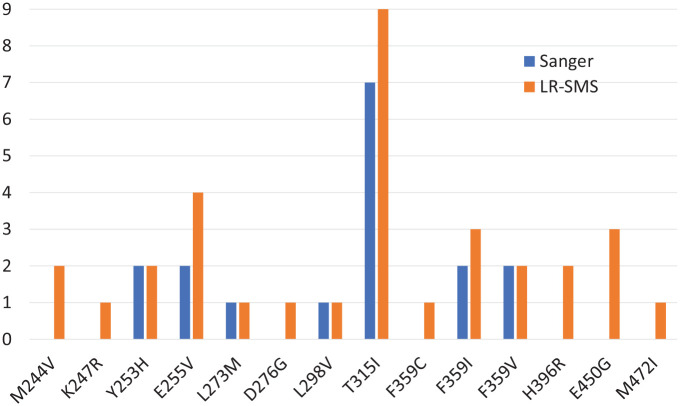
Mutations found in the samples of this study. Number samples where each mutation is found by Sanger or long-read sequencing. Mutations: M244V secondary resistance^8^; K247R rare, natural^16^; Y253H secondary resistance^8^; E255V secondary resistance^8^; L273M rare, uncertain^17^; D276G rare, uncertain^18^; L298V rare, uncertain^17^; T315I secondary resistance^8^; F359C secondary resistance^8^; F359I secondary resistance^8^; F359V secondary resistance^8^; H396R secondary resistance^19^; E450G unknown^15^; M472I unknown.^20^

In Samples 1, 12, 14, 24, and 29, LR-SMS found the Sanger mutations but also reported others, undetected by Sanger. These additional mutations were near or below Sanger’s detection limit but could still be of clinical significance (see Figure 5). In some samples, secondary resistance mutations E255V, F359C, H396R, and even the common T315I were found only by LR-SMS.

In Sample 33, both methods found the 2 mutations L298V and E255K. The methods agreed on mutation T315I for Samples 10 and 16, with values approaching 100% for LR-SMS. Sample 2 showed mutations F359I and T315I with both methods.

In Samples 3, 5, 18, 26, 28 and 35, LR-SMS found mutations where Sanger reported only wild-type. In these samples, LR-SMS reported mutation levels well below Sanger’s practical detection limit but these were also below the 1% limit considered relevant for the clinic. Some of these low-level mutations are associated with resistance (T315I and M244V) and others are of uncertain significance (E450G, M472I).

In summary, LR-SMS discovered mutations which were not found by Sanger. All (9) samples with Sanger reported mutations show (at least) these same mutations with LR- SMS. All mutations found by Sanger were also found by LR-SMS. At a conservative cutoff of 5% for LR-SMS, 5 samples showed fewer or no mutations with Sanger. At the practical detection limit for LR-SMS,^12^ 15 samples showed fewer or no mutations with Sanger. Overall, LR-SMS found every mutation reported by Sanger sequencing and revealed many more missed by Sanger.

After these encouraging results, the method was further validated and eventually introduced into clinical routine. Since 2015, our implementation has been validated twice every year in the UK-NEQAS program “BCR::ABL1 Kinase Domain Variant Mutation Status.” UK-NEQAS performs independent monitoring of laboratory tests, and is used by many clinical labs in Europe to ensure the quality of their accredited analyses (https://ukneqas.org.uk). Within this program, control samples with mutations at specific sites in the BCR-ABL1 transcript were supplied by UK-NEQAS to us and to other clinical laboratories across Europe. After having reported our results back to UK-NEQAS, we can conclude that our analysis has obtained a 100% success rate in all test rounds. This means that all true positive mutations have been found, while no false positives mutations have been reported, in any of the analyzed control samples. We are thus confident that our analysis method has an acceptable false positive rate and meets all requirements for clinical reporting.

## Implications

Clinical laboratories are in many cases interested in using the latest technologies if it can lead to improved diagnoses and treatments, while not drastically increasing turn-around time or costs. However, it is generally a difficult and slow process to integrate new technologies into clinical practice, and most technologies run at hospitals are mature and have been proven over several years. For long-read sequencing, there is also an added component needed in the form of computational resources and expertise in (bioinformatics) data analysis. If the entire data analysis is not automated, it presents additional challenges to incorporate in clinical practice.

New technological developments and applications can be found in academic settings, such as in core facilities serving the scientific community. It is not uncommon that these technologies mature over a period of years, and ultimately make their way into the clinic. In our work, we bridged the gap and allowed Uppsala University Hospital to make use of the academic sequencing facility for performing long-read sequencing. The main components to enable this were processes to prepare and exchange samples between the clinic and the facility, automated workflows for the sequence analysis, and a system for storing and reporting results back to clinicians for interpretation. One of the keys to helping clinicians switch over to a new technology was to get their input in the design process of reports and interfaces.

In this manuscript we present the first fully integrated solutions for long-read sequencing comprising the complete workflow, from sample registration via long-read sequencing at a core facility to clinical decision aid on the hospital side, to detect low-frequency mutations in high coverage long-read amplicon data, such as in this BCR-ABL1 project. Although it would have been possible to perform alignment using standard tools such as minimap2 followed by variant calling by DeepVariant, it is important to note that such analyses have been developed for germline variation but have not been optimized for detection of low-frequency somatic variation. Since many of the variant calling tools for long-read are based on machine learning, it would be a non-trivial task to apply these for somatic variant calling and with uncertain outcomes. Moreover, our analysis strategy contains specific steps fine-tuned for the BCR-ABL1 region; for example, filtering is performed in order to only analyze reads originating from the BCR-ABL1 molecules while filtering out reads originating from ABL1 (not fused with BCR). We also perform specific quality control steps to ensure that all clinically relevant mutation positions can be assigned either as a negative or a positive call. To the best of our knowledge, no other existing pipelines can detect cancer mutations down to a frequency of 1% from the long-read data generated within this project.

The possibility to take advantage of long-read sequencing via a core facility includes no up-front costs for sequencing instruments, which currently are quite expensive, at least for PacBio SMRT sequencing. Further, costs are kept lower as utilization of sequencing instruments is higher when shared with academic researchers. Finally, the clinical organization is not required to build up and maintain state-of- the-art expertise in bioinformatics. The information system developed in this project can be directly reused to serve as a blueprint for other long-read sequencing applications.

## Conclusion

We present an example where long-read sequencing was implemented for BCR-ABL1 TKI resistance mutation screening, as a clinical routine analysis. The process comprised linking the clinic to an academic sequencing facility where sequencing of samples was carried out, the development of a supportive information system, and validation steps to verify the methodology. Key components in the translation were the processes for sample preparation and transfer, automated data analysis pipelines, and a shared system for storage and reporting. Long-read sequencing was shown to have advantages over previous methods, such as higher sensitivity, and ultimately replaced Sanger sequencing as the routine method for detection of BCR-ABL1 mutations.

## Supplemental Material

sj-pdf-1-cix-10.1177_11769351221110872 – Supplemental material for Migrating to Long-Read Sequencing for Clinical Routine BCR-ABL1 TKI Resistance Mutation Screening Click here for additional data file. Supplemental material, sj-pdf-1-cix-10.1177_11769351221110872 for Migrating to Long-Read Sequencing for Clinical Routine BCR-ABL1 TKI Resistance Mutation Screening by Wesley Schaal, Adam Ameur, Ulla Olsson-Strömberg, Monica Hermanson, Lucia Cavelier and Ola Spjuth in Cancer Informatics

sj-pdf-2-cix-10.1177_11769351221110872 – Supplemental material for Migrating to Long-Read Sequencing for Clinical Routine BCR-ABL1 TKI Resistance Mutation Screening Click here for additional data file. Supplemental material, sj-pdf-2-cix-10.1177_11769351221110872 for Migrating to Long-Read Sequencing for Clinical Routine BCR-ABL1 TKI Resistance Mutation Screening by Wesley Schaal, Adam Ameur, Ulla Olsson-Strömberg, Monica Hermanson, Lucia Cavelier and Ola Spjuth in Cancer Informatics

sj-pdf-3-cix-10.1177_11769351221110872 – Supplemental material for Migrating to Long-Read Sequencing for Clinical Routine BCR-ABL1 TKI Resistance Mutation Screening Click here for additional data file. Supplemental material, sj-pdf-3-cix-10.1177_11769351221110872 for Migrating to Long-Read Sequencing for Clinical Routine BCR-ABL1 TKI Resistance Mutation Screening by Wesley Schaal, Adam Ameur, Ulla Olsson-Strömberg, Monica Hermanson, Lucia Cavelier and Ola Spjuth in Cancer Informatics

sj-pdf-4-cix-10.1177_11769351221110872 – Supplemental material for Migrating to Long-Read Sequencing for Clinical Routine BCR-ABL1 TKI Resistance Mutation Screening Click here for additional data file. Supplemental material, sj-pdf-4-cix-10.1177_11769351221110872 for Migrating to Long-Read Sequencing for Clinical Routine BCR-ABL1 TKI Resistance Mutation Screening by Wesley Schaal, Adam Ameur, Ulla Olsson-Strömberg, Monica Hermanson, Lucia Cavelier and Ola Spjuth in Cancer Informatics

sj-pdf-5-cix-10.1177_11769351221110872 – Supplemental material for Migrating to Long-Read Sequencing for Clinical Routine BCR-ABL1 TKI Resistance Mutation Screening Click here for additional data file. Supplemental material, sj-pdf-5-cix-10.1177_11769351221110872 for Migrating to Long-Read Sequencing for Clinical Routine BCR-ABL1 TKI Resistance Mutation Screening by Wesley Schaal, Adam Ameur, Ulla Olsson-Strömberg, Monica Hermanson, Lucia Cavelier and Ola Spjuth in Cancer Informatics
