# Supplementary material for: Migrating to Long-Read Sequencing for Clinical Routine BCR-ABL1 TKI Resistance Mutation Screening
Source: Cancer Inform. 2022 Jul 15;21:11769351221110872. doi: 10.1177/11769351221110872 (PMC9290162; doi:10.1177/11769351221110872)
Supplement: sj-pdf-1-cix-10.1177_11769351221110872 – Supplemental material for Migrating to Long-Read Sequencing for Clinical Routine BCR-ABL1 TKI Resistance Mutation Screening [file sj-pdf-1-cix-10.1177_11769351221110872.pdf]

Workflow for PacBio BCR-ABL1 mutation screening

Step 1. Register samples in text file and in the web system

As soon as new samples have been delivered, update the sample information in the file 'bcr-abl1_sample_list_with_primers.txt'. Go to the clamp directory and open the file in your favorite text editor.

Add all necessary fields (runId, sampleId, primerId, site and date) for each of the new samples to be analyzed. The fields must be separated by tab '\t' characters.

1. *runID*: Unique identifier related to the sequencing run
2. *sampleId*: Identifier from clinic. Unique for the sample but possible run multiple times
3. *primerId*: Name of a primaer found in 'target_info.txt'. Note spelling.
4. *site*: Short abbreviation for the source of the sample.
5. *date*: yyy-mm-dd

Login to your CLAMP server. Click on 'Admin' link, then 'Register Samples'. Copy/paste all the newly added sample rows from 'bcr-abl1_sample_list_with_primers.txt' into the form and click on the box 'check'. If no problems occurred in the naming of samples click 'save' on the next page.

Step 2. Run 'reads of insert' plugin in SMRT portal

When the PacBio run is complete, log on to the SMRT portal using your personal user ID. Click on 'Create New' and select the SMRT cell containing the data for the patient to be analyzed. Select 'RS_ReadsOfInsert.1' under the 'Protocols', assign a job name for the analysis (eg, 'foo_002_1_ccs_reads') and click on the 'Start' button.

Step 3. Store result files on a local computer

Create a new directory for the runID which matches the filename above (eg, 'foo_002_1').

When the 'RS_ReadsOfInsert.1' plugin is done, download the FASTQ file to the newly created directory. The FASTQ file should then be renamed so that the end with 'fastq', not 'fastq.txt'. Make certain common file extensions aren't hidden.

Step 4. Run the BCR-ABL1 mutation screening analysis

Go to the directory 'clamp' and start R.

Step 5. Perform QC on the run and analysis

In R, run the program 'clamp_bcr-abl1.R'

```
> source("clamp_bcr-abl1.R")
> screenMutationsCML()
```

This will process all new samples in the 'bcr-abl1_sample_list_with_primers.txt' file. A number of files will be created in the run directory for each sample. Running the same ls -l command as in the example above now gives:

```
igp-47-94:foo_002_1 adame789$ ll
total 313072
```

```
-rw-rw----  1 adame789  staff   1.7K Oct 25 13:36 foo_002_1_analysis_l
-rw-rw----  1 adame789  staff   119K Oct 25 13:36 foo_002_1_bcr-13-abl
-rw-rw----  1 adame789  staff    13K Oct 25 13:36 foo_002_1_bcr-14-abl
-rw-rw----  1 adame789  staff   4.5K Oct 25 13:36 foo_002_1_bcr-14-abl
-rw-rw----  1 adame789  staff   407B Oct 25 13:36 foo_002_1_bcr-14-abl
-rw-rw----  1 adame789  staff   788B Oct 25 13:36 foo_002_1_bcr-14-abl
-rw-rw----  1 adame789  staff   126B Oct 25 13:36 foo_002_1_bcr-14-abl
-rw-rw----  1 adame789  staff   4.4M Oct 25 13:36 foo_002_1_bcr-14-abl
-rw-rw----  1 adame789  staff   4.6M Oct 25 13:36 foo_002_1_bcr-14-abl
-rw-rw----  1 adame789  staff   4.6M Oct 25 13:36 foo_002_1_bcr-14-abl
-rw-rw----  1 adame789  staff   4.8M Oct 25 13:36 foo_002_1_bcr-14-abl
-rw-rw----  1 adame789  staff   5.0M Oct 25 13:36 foo_002_1_bcr-14-abl
-rw-rw----  1 adame789  staff   7.1K Oct 25 13:36 foo_002_1_bcr-14-abl
-rw-rw----  1 adame789  staff   8.6K Oct 25 13:36 foo_002_1_bcr-14-abl
-rw-rw----  1 adame789  staff   133K Oct 25 13:36 foo_002_1_bcr-14-abl
-rw-rw----  1 adame789  staff   125K Oct 25 13:36 foo_002_1_bcr-14-abl
-rw-rw----  1 adame789  staff    15K Oct 25 13:36 foo_002_1_candidate_
-rw-rw----  1 adame789  staff   549K Oct 25 13:36 foo_002_1_identical_
-rw-r-----  1 adame789  staff    33M Oct 25 13:36 foo_002_1_reads_of_i
-rw-rw----  1 adame789  staff    96M Oct 25 13:37 foo_002_1_reads_of_i
```

Unless any files with prefix 'QC_FAILED_' are found in the directory, most of the mutations were screened high coverage and it is possible to proceed to Step 6. If a 'QC_FAILED_' file exists a more thorough troubleshooting is required, but the likely explanation is that insufficient coverage was obtained and the sample needs to be rerun.

Step 6. Quality control of coverage data

To assess the quality of the sequencing data, open the quality control pdf (...QC.pdf) file and look at the coverage profile. If there is a continuous coverage profile over the whole sequence and the minimum coverage (represented by the red horizontal line) is above 100, then the sample has passed the quality control criteria. If there are any drops in coverage, a more detailed analysis is required.

Step 7. Detection of previously unknown mutations

The system automatically screens for previously unknown mutations occurring at a frequency of at least 2.5% among the 500 highest quality reads. If the file 'denovo_snps.txt' exists, then look in to the file. One example of such a file for sample foo_002_1 is shown below:

```
sequence        pos     ref     alt     cov_fwd ref_fwd alt_fwd cov_re
GCCCCCGTTCTATATCATCA[C/T]TGAGTTCATGACCTACGGGA   1002    C       T
...
```

If there is any row that has a 'NA' in the annotation field, then this corresponds to a potential novel mutation. If such a novel mutation is detected, a more detailed investigation is mutation position is needed before uploading the final results. Otherwise, proceed to Step 8.

Step 8. Upload result files into web based system

Go back to your CLAMP server and upload the results. Once logged in to the system, click the 'Admin' link and then 'Upload Files'. Select the mutation results text file (suffix final.txt), the raw reads file (fastq.gz), the quality control file (QC.pdf) and the log file (analysis_log.txt). If a clonal distribution analysis was performed, two additional files will be created (clonal_distribution.txt and clonal_distribution.pdf). Both these files should also be uploaded in the system. Once all files have been selected, click the submit button and the results are be imported into the system!

Step 9. Send a mail to inform that mutation results are ready!

Inform everyone in the group that new results are ready. Send a mail using the template below:

```
Subject:
[CML PacBio] BCR-ABL1 mutation analysis of samples - week XX yyyy



Message:
New BCR-ABL1 mutation results are ready (https://<clamp-server>/). Kno
and previously unknown mutations of at least 5% have been screened in
is required at a mutational position in order to class it as positive
lower than 100 are classed as unresolved.
*** samples ***
run_id_1 sample_id_1
run_id_2 sample_id_2
```
