# Supplementary material for: Migrating to Long-Read Sequencing for Clinical Routine BCR-ABL1 TKI Resistance Mutation Screening
Source: Cancer Inform. 2022 Jul 15;21:11769351221110872. doi: 10.1177/11769351221110872 (PMC9290162; doi:10.1177/11769351221110872)
Supplement: sj-pdf-2-cix-10.1177_11769351221110872 – Supplemental material for Migrating to Long-Read Sequencing for Clinical Routine BCR-ABL1 TKI Resistance Mutation Screening [file sj-pdf-2-cix-10.1177_11769351221110872.pdf]

Usage notes for retreiving results (GUI for technicians)

Data registration and upload is done through the Admin Page. This is the sample meta-data which is kept separate from the calculation results. As suggested in the GUI installation notes, it's possible that access is restricted (beyond the general server restrictions).

Registration

Register samples for Philadelphia Variants

- Register Samples
- Upload Files

Registration, as described in step 1 of the Calc usage document, is the mechanism to add a sample's meta-data. The registration page is simply an open text field. This allows technicians to more easily copy/paste from local documents and to be more flexible when new fields are needed. The form accepts multiple records (one per line) with fileds separated by spaces and/or tabs.

Run Id	Sample Id	Primer	Site #	Date

After clicking the Check button, a second screen appears where you can make certain that the data have been interpreted as expected. The entries are color-coded as follows:

- Samples already in the database are shown here with a grey background. These will be skipped if you try to save.
- Uninterpretable or missing fields will be marked with a red background. These will be skipped if you try to save.
- Suspicious fields will be marked with a green background. These are probably wrong but can be saved if you know they are correct.
- New and completely normal-looking entries will have no background color. These, of course, can be saved.

Clicking Save on this page will add the elligible records to the database.

Uploading

Uploading results is described in step 8 of the Calc usage document. The current version of Clamp has a rather cumbersome system to upload the several files all individually. This is partially for historical reasons (we decided to store more files) and for flexibility (a subset of files can be uploaded depending on circumstances). More automated options have been explored but are not shown here.

Upload data files for registered samples

File names determine the "Run ID" they will be associated with. Use caution.
All files are technically optional but it's rather boring without "Results".

Results file (final.txt) Choose File no file selected	Sequences (.fastq.gz) Choose File no file selected	Coverage (QC.pdf) Choose File no file selected
Clonal Distribution (.txt) Choose File no file selected	Clonal Distribution (.pdf) Choose File no file selected	Log file (log.txt) Choose File no file selected
Filtered (filtered.fastq) Choose File no file selected		

Submit Reset

Uploaded files are stored in a file system with the paths recorded in the database. The files belonging to a sample are grouped into a folder named after the run ID. Each application (instance) retains it's own group of files (possibly organized with symbolic links).

Note that Clamp does not specifically include a backup strategy. We make scheduled rsync dumps to an external system.
