# Supplementary material for: Migrating to Long-Read Sequencing for Clinical Routine BCR-ABL1 TKI Resistance Mutation Screening
Source: Cancer Inform. 2022 Jul 15;21:11769351221110872. doi: 10.1177/11769351221110872 (PMC9290162; doi:10.1177/11769351221110872)
Supplement: sj-pdf-3-cix-10.1177_11769351221110872 – Supplemental material for Migrating to Long-Read Sequencing for Clinical Routine BCR-ABL1 TKI Resistance Mutation Screening [file sj-pdf-3-cix-10.1177_11769351221110872.pdf]

Software installation for storage and reporting frontend

Requirements

- Apache 2.4
    - must be cgi enabled (serve-cgi-bin.conf)

Some other webserver could be used but the file organization and security model for CLAMP were design to fit into a pre-existing server running a variety of applications. An example httpd.conf follows (where "path/to..." should obviously be localized).

```
<Directory path/to/cml>
AuthName Foo
AuthType Basic
AuthUserFile path/to/passwd
AuthGroupFile path/to/passwd/group
AddHandler cgi-script .cgi
Options +Indexes +ExecCGI +FollowSymLinks
DirectoryIndex index.cgi
AllowOverride Limit
Require valid-user
</Directory>

<Directory path/to/cml/reg>
Require group cml
</Directory>
```

The *password* file looks like:

```
alice:<encrypted>
bob:<encrypted>
carol:<encrypted>
```

The *group* file looks like:

```
cml: bob carol
```

So, in this example, Bob and Carol would be able to register and upload samples. Alice would only be allowed to search and view the results.

- Perl, eg version 5.22 but at least 5.10
    - Perl Modules (with minimum or suggested version):
    - CGI 4.26
    - CGI::Carp 4.26
    - Compress::Zlib 2.068
    - DBD::SQLite 1.56
    - File::Copy 2.30
    - GD 2.53
    - GD::Graph 1.54

- GD::Text 0.86
- List::Util 1.41

Note that some of these Perl modules are or have been in Perl core. Some modules may have external dependencies (most notably *GD*). The listed version numbers are known to work but are unlikely to be strictly required.

The DBD::SQLite module holds a working copy of SQLite (version 3.22 in this case) so a separate installation isn't strictly necessary to run the system.

Local configuration

An *uploads* folder must be visible to the scripts for the actual uploading as well as file downloads. This is probably handled with symbolic links and may involve remote storage since a single processed sample may be 500 MB.

A *data* folder containing a SQLite database. The database should be created with a version of SQLite compatible with the DBD module used above. Note that MySQL was used in the past and some files have the connection code commented out for easy reference.

The table schema are as follows:

```
CREATE TABLE files (
seqid INTEGER PRIMARY KEY AUTOINCREMENT,
regid integer, versn integer, orig, ftype, ruser,
rhost,stamp timestamp DEFAULT CURRENT_TIMESTAMP);

CREATE TABLE logs (regid integer not null, date, type, ref);

CREATE TABLE results (
regid integer not null, sortby integer not null, mutation, sequence,
wt_reads_fwd, mut_reads_fwd, other_reads_fwd, freq_fwd, wt_reads_rev,
mut_reads_rev, other_reads_rev, freq_rev, wt_reads, mut_reads,
other_reads, freq, detection, routine);

CREATE TABLE ontarget (regid integer not null, filtered, raw, ontarg);

CREATE TABLE samples (
regid INTEGER PRIMARY KEY AUTOINCREMENT,
runid, samid, primr, srcid, sdate, ispnt, notes, ruser, rhost,
stamp timestamp DEFAULT CURRENT_TIMESTAMP);

CREATE TABLE primers (primerId, assay, fwd, rev);
```
