# Supplementary material for: Migrating to Long-Read Sequencing for Clinical Routine BCR-ABL1 TKI Resistance Mutation Screening
Source: Cancer Inform. 2022 Jul 15;21:11769351221110872. doi: 10.1177/11769351221110872 (PMC9290162; doi:10.1177/11769351221110872)

Usage notes for retreiving results (GUI for clinicians)

Clamp users start with a blank search screen:

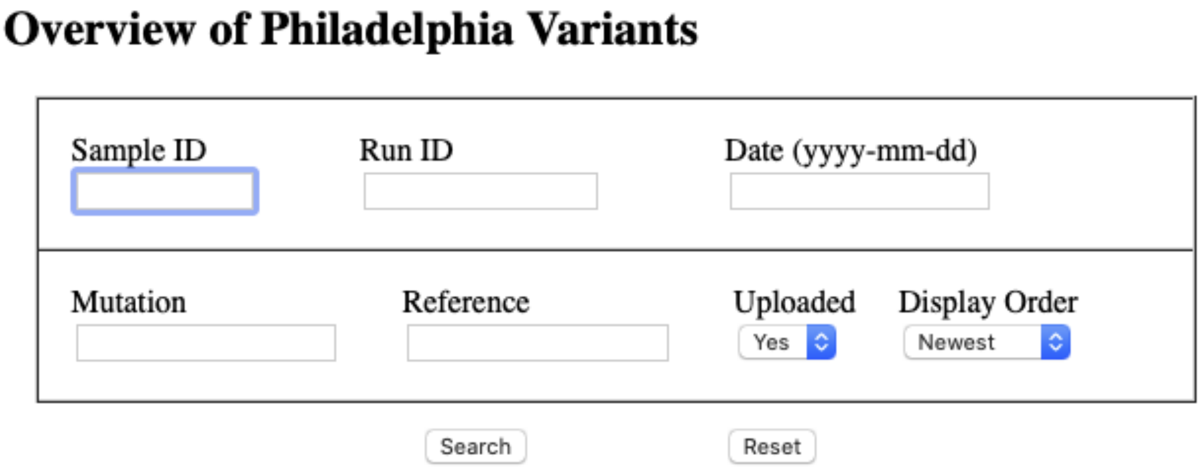

Values can be entered into these fields to limit the results. In the image below, the search has been limited to Run IDs starting with "foo_002" (note the use of "*" as a wildcard) and a Date greater than 2020-01-01 (note the use of ">" as a comparator and yyy-mm-dd format).

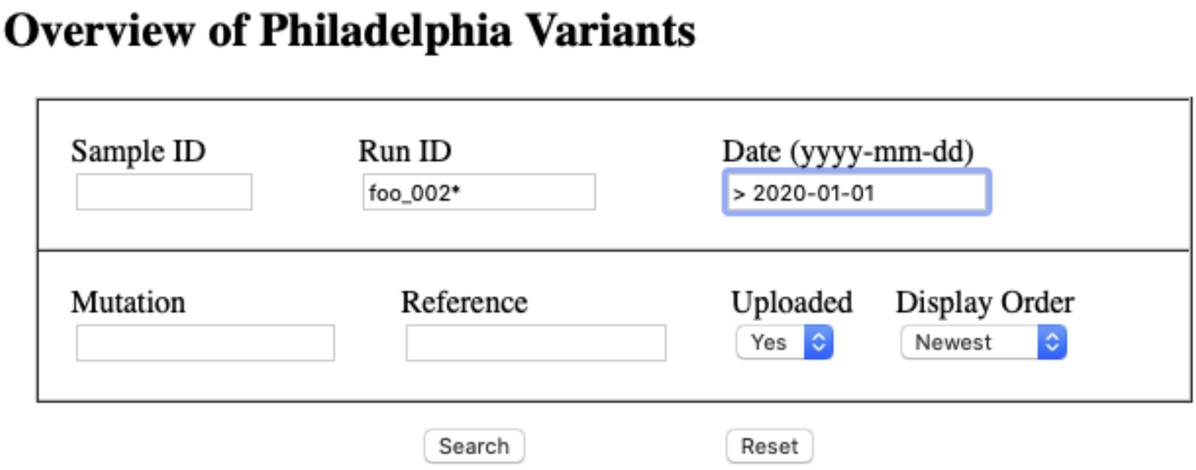

After clicking *Search*, a list of hits will appear, showing the calculated percentages of any any mutations found. Some fields have been omitted here for clarity.

Sample	Unresolved	M244V	Y253H	E255V	D276G	T315I	F359C	F359I	H396R
1					2	96.8	2.1		1.2
2						14.4		84.9	
3		0.6							
4			98.1	1.7					
5		0.9							
6									

Clicking on the numbers in the left-most column will bring up a detailed view for that sample including access to raw data files. Details unavailable to the current sample (eg, no clonal distribution for samples with fewer than ywo mutations). The navigation panel at the top can be used to view other selected samples.

---

5 **Sample 6** 7 New Search

Sample ID	Run ID	Date
[redacted]		

Downloads:

Results Sequence Coverage Clonal txt Clonal pdf Log

OnTarget%	Background
61.5 %	0.001

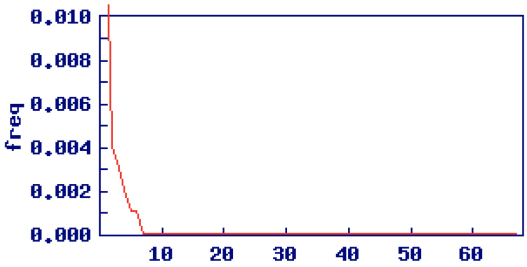

Supplement: sj-pdf-4-cix-10.1177_11769351221110872 – Supplemental material for Migrating to Long-Read Sequencing for Clinical Routine BCR-ABL1 TKI Resistance Mutation Screening [file sj-pdf-4-cix-10.1177_11769351221110872.pdf]
