# Supplementary material for: Migrating to Long-Read Sequencing for Clinical Routine BCR-ABL1 TKI Resistance Mutation Screening
Source: Cancer Inform. 2022 Jul 15;21:11769351221110872. doi: 10.1177/11769351221110872 (PMC9290162; doi:10.1177/11769351221110872)
Supplement: sj-pdf-5-cix-10.1177_11769351221110872 – Supplemental material for Migrating to Long-Read Sequencing for Clinical Routine BCR-ABL1 TKI Resistance Mutation Screening [file sj-pdf-5-cix-10.1177_11769351221110872.pdf]

CLAMP

Automated analysis pipeline for long-read sequencing and an information system to store and present results. These tools are focused on use in a clinical setting and are currently applied to *BCR-ABL1* TKI resistance mutation screening for patients undergoing treatment for chronic myeloid leukemia and related diseases.

CLAMP consists of separate parts for sequence data processing (clamp-calc) and informatics (clamp-gui). While designed to work in unison, installation and usage will be explained separately since they are possibly installed on separate systmes and used by different people.

Data processing:

- Calc Installation
- Calc Processing
- Reporting

Results:

- GUI Installation
- GUI usage

Copyright 2022 Pincer Bio AB
CLAMP is available under the terms of Apache 2.0 Licence.

A manuscript is in progress:
"Migrating to Long-Read Sequencing for Clinical Routine *BCR-ABL1* TKI Resistance Mutation Screening"
Wesley Schaal, Adam Ameur, Ulla Olsson-Strömberg, Monica Hermanson, Lucia Cavelier and Ola Spjuth
